# Phage vB_BveM-Goe7 represents a new genus in the subfamily *Bastillevirinae*

**DOI:** 10.1007/s00705-020-04546-1

**Published:** 2020-02-12

**Authors:** Alexandra Dominique Furrer, Mechthild Bömeke, Michael Hoppert, Robert Hertel

**Affiliations:** 1 Department of Genomic and Applied Microbiology & Göttingen Genomics Laboratory, Institute of Microbiology and Genetics, Georg-August-University Göttingen, Göttingen, Germany; 2 Department of General Microbiology, Institute of Microbiology and Genetics, Georg-August-University Göttingen, Göttingen, Germany

## Abstract

*Bacillus velezensis* FZB42 is a Gram-positive, endospore-forming rhizobacterium that is associated with plant roots and promotes plant growth. It was used as host to isolate phage vB_BveM-Goe7 (Goe7). Goe7 exhibits a *Myoviridae* morphology with a contractile tail and an icosahedral head. Its genome is 158,674 bp in size and contains 5137-bp-long terminal repeats (LTRs). It also contains five tRNA-encoding genes and 251 coding DNA sequences (CDS), of which 65 were annotated. The adsorption constant of Goe7 is 6.1 ± 0.24 × 10^−8^ ml/min, with a latency period of 75 min and a burst size of 114 particles per burst. A BLASTn sequence comparison against the non-redundant nucleotide database of NCBI revealed that Goe7 is most similar to *Bacillus subtilis* phage vB_BsuM-Goe3.

**Electronic supplementary material:**

The online version of this article (10.1007/s00705-020-04546-1) contains supplementary material, which is available to authorized users.

*Bacillus velezensis* FZB42 is a rod-shaped aerobic soil bacterium [1]. This member of the *B.* *subtilis* species complex [2] was originally proposed as the type strain of *Bacillus amyloliquefaciens* subsp*. plantarum* before it was recognized as *B.* *velezensis* [3]. Many strains of this group are root-associated and are able to promote plant growth [4]. We used strain FZB42 as host for the isolation of phage vB_BveM-Goe7 (Goe7) from the Göttingen municipal sewage plant (Göttingen, Germany, 51° 33′ 15.4′′ N 9°55′06.4′′ E). For this, an overlay plaque assay was used. Goe7 could be isolated as a clear plaque in the bacterial layer, indicating a lytic life style. The morphology of Goe7 was determined via electron microscopy, using a Jeol 1101 electron microscope (Eching, Munich, Germany) as described previously [5]. TEM micrographs revealed a *Caudovirales*-related head-tail morphology (Fig. 1) and a *Myoviridae*-like contractile tail (Fig. 1A) [6]. A double baseplate upon tail contraction (Fig. 1A) indicates a morphological relatedness to the SPO1-like phages [7]. The icosahedral head is 88.2 nm ± 4 nm in diameter and the tail is 216 nm ± 10.4 nm long and 16.4 nm ± 1.9 nm wide.

**Fig. 1:**
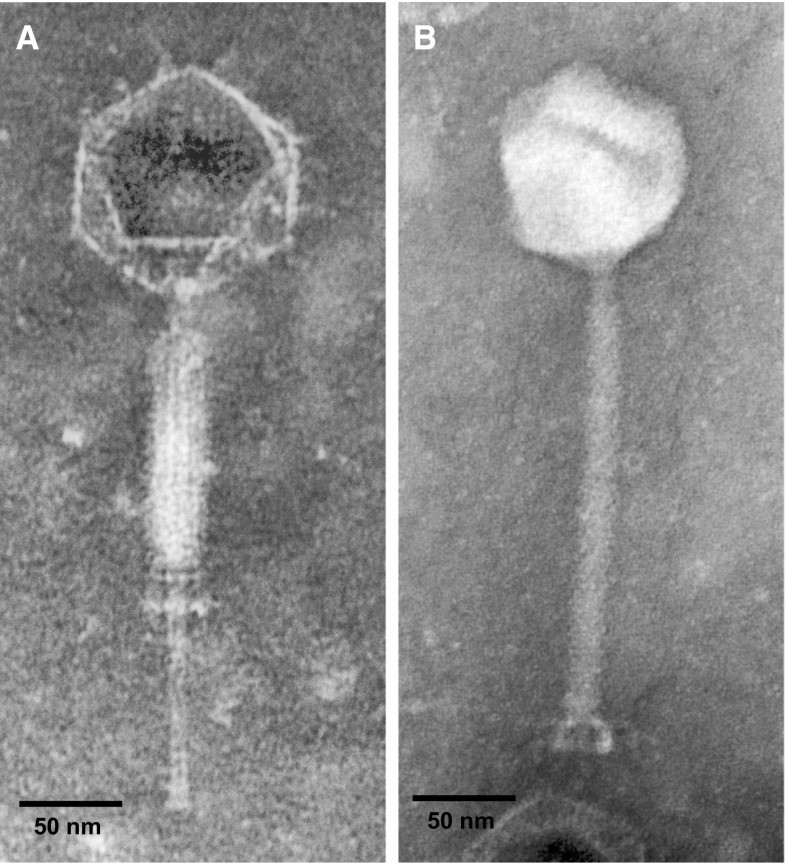
vB_BveM-Goe7 micrographs. (A) Goe7 with contracted tail sheath. The baseplate reveals a double stack structure. (B) Complete infectious Goe7 particle

Genome sequencing and quality assessment of the obtained sequence reads were done as described previously [5]. Initial assembly was performed with SPAdes version 3.10.1 [8]. The ends of the long terminal repeats (LTR) were resolved via Sanger sequencing as described for phage vB_BsuM-Goe2 (Goe2) and VB_BsuM-Goe3 (Goe3) [9] using the primers Goe3_P4 (5’GCGGTATGTCTGAATAAGGG) and PP004 (5’GCACATGACAGGGATTCAAC).

The genome of Goe7 consists of a linear, double-stranded DNA chromosome with a size of 158,674 bp and a GC-content of 41.84%. Automatic annotation using the Prokka pipeline [10] identified five tRNA-encoding genes and 251 coding DNA sequence (CDS). A domain search using InterProScan [11] (Supplemental Table S1) was used to improve the initial annotation of Prokka and led to the annotation of 65 protein coding genes (Supplemental Table S2). About three quarters of the protein genes remained hypothetical, indicating hitherto unknown genes and functions.

BLASTn comparison of Goe7 with the non-redundant nucleotide database of NCBI revealed the highest similarity to *Bacillus* phage vB_BsuM-Goe3 [9]. It showed 96% query coverage with 98.92% identity. Further related phages with a query coverage of >20% were *Bacillus* phage BSP38 (MH606185.1) [12], SBSphiJ (LT960608.1), Grass (KF669652.1) [13] and phiNIT1 (AP013029.1), all of which are members of the lytic group of SPO1-related phages [7]. Average nucleotide identity (ANI) values [14] were calculated using the average_nucleotide_identity.py (https://github.com/widdowquinn/pyani) script with the ANIm option employing MUMer3 [15]. The genome sequence of Goe7 was compared with those of members of the phage family *Herelleviridae*, which infect members of the genus *Bacillus* [16]. The results revealed Goe7 to be a unique isolate, with 98.18% overall genome sequence identity to *B.* *subtilis* phage Goe3, indicating that it belongs to the same species (Fig. 2 and Supplemental Table S3). ANIm alignment lengths (Supplemental Table S4) perfectly reassembled all genera of the subfamilies *Bastillevirinae* and *Spounavirinae* as proposed by Barylski *et al.* [16]. In this context it becomes evident that Goe7 and Goe3 form a separate genus. This new genus fits into the subfamily *Bastillevirinae*, as Goe7 shows similarities to members of the genera *Agatevirus* (Bobb 86.7% and Bp8p 87.1%) and *Nitunavirus* (Grass 84.1%, SPG24 83.7%, phiNIT1 83.1%) (Supplemental Table S3).

**Fig. 2:**
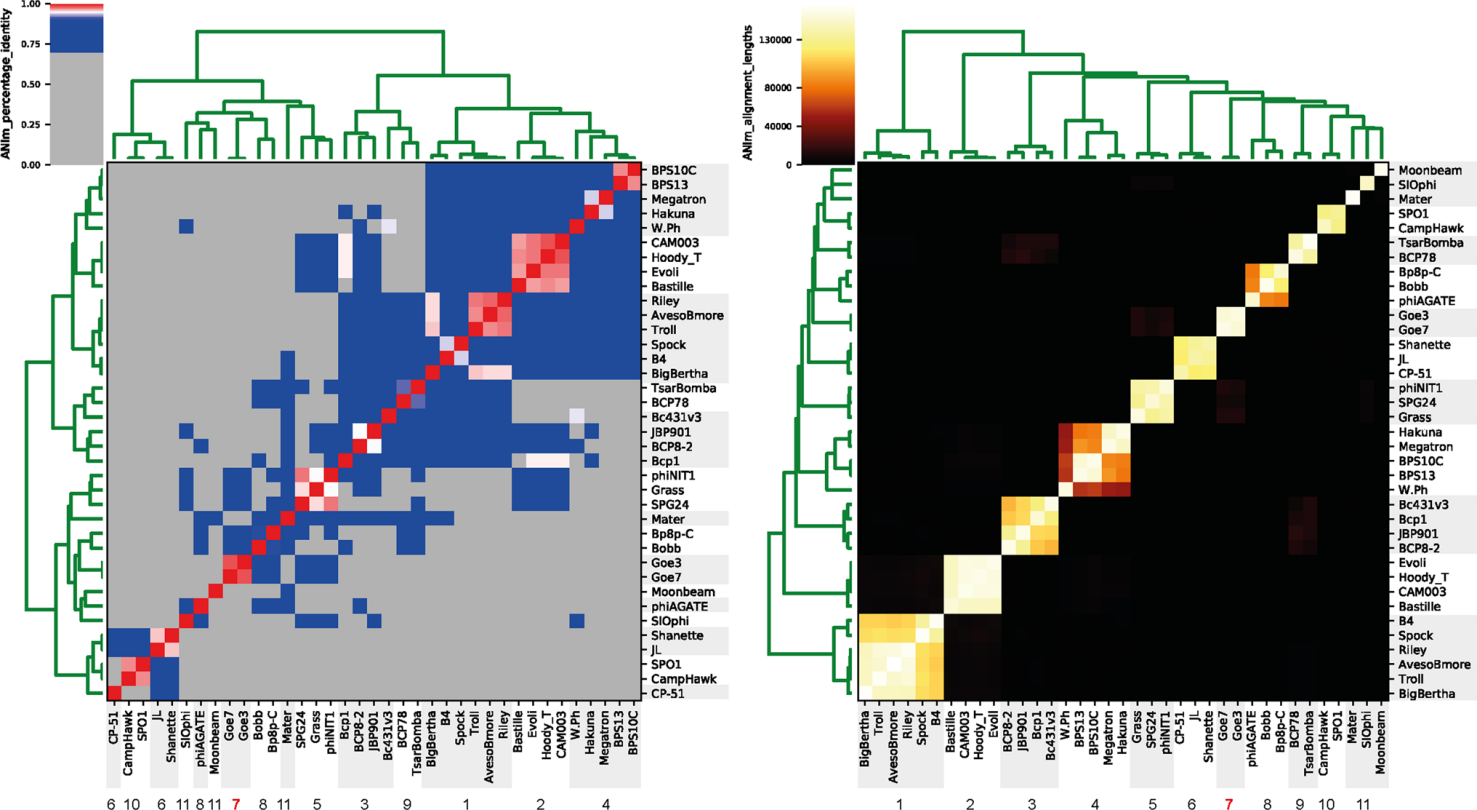
Average nucleotide identity and alignment length analysis of Goe7. Genera as described by Barylski et al. [16] were used for this investigation and are indicated with shaded gray bars (1 = *Bequatrovirus*, 2 = *Bastillevirus*, 3 = *Caeruleovirus*, 4 = *Wphvirus*, 5 = *Nitunavirus*, 6= *Siminovitchvirus*, 8 = *Agatevirus*, 9 = *Tsarbombavirus*, 10 = *Okubovirus*, 11 = recently unassigned strains). The average nucleotide identity values were calculated with the ANIm option. Percentage identity values are visualized on the left. A reddish color indicates the same species with an ANI value >95%. White indicates an ANI value of ~95% and the species boundary. A bluish color indicates an ANI value of 70–95% and a high degree of relatedness. Alignment lengths are visualized on the right. The brighter the color from black over red and yellow to white, the longer is the related alignment. Goe7 is of the same species as Goe3 due to 98% genome identity (left). Both strains form a new genus (**7**), as indicated by the alignment length of the ANIm analysis (right)

The adsorption constant of Goe7 is 6.1 × 0.24^−8^ ml/min and was determined as described by A. M. Kropinski [17]. A latency period of 75 min and a burst size of 114 particles per burst were observed in a one-step growth experiment carried out as described by Hyman and Abedon [18] in a 250-ml conical flask with 25 ml of LB with vigorous shaking at 30 °C (see Table S5). Goe3, which infects *B.* *subtilis*, a close relative of *B.* *velezensis*, differs from Goe7 in its effect on its host [9]. Although Goe7 has an identical burst size (114 particles per burst), it adsorbs more efficiently to its host than Goe3 (adsorption constant, 8 × 10^−11^ ml/min) and requires more time for replication than Goe3 latency period (55 min).

The final genome sequence of Goe7 is publicly available in the GenBank database with the accession number MN043730. Biological samples of vB_BveM-Goe7 with the sample number DSM109177 are available in the German Collection of Microorganisms and Cell Cultures (DSMZ).

## Electronic supplementary material

Below is the link to the electronic supplementary material.

Supplementary material 1 (XLSX 86 kb)

## References

[1] Chen XH, Koumoutsi A, Scholz R, Eisenreich A, Schneider K, Heinemeyer I, Morgenstern B, Voss B, Hess WR, Reva O (2007). Comparative analysis of the complete genome sequence of the plant growth-promoting bacterium *Bacillus amyloliquefaciens* FZB42. Nat. Biotechnol..

[2] Fan B, Blom J, Klenk HP, Borriss R (2017). *Bacillus amyloliquefaciens*, *Bacillus velezensis*, and *Bacillus siamensis* form an “Operational Group *B. amyloliquefaciens*” within the *B. subtilis* species complex. Front Microbiol.

[3] Dunlap CA, Kim S-J, Kwon S-W, Rooney AP (2016). *Bacillus velezensis* is not a later heterotypic synonym of *Bacillus amyloliquefaciens*; *Bacillus methylotrophicus*, *Bacillus amyloliquefaciens* subsp. plantarum and ‘*Bacillus oryzicola*’ are later heterotypic synonyms of *Bacillus velezensis* based on phylogenom. Int J Syst Evol Microbiol.

[4] Fan B, Wang C, Song X, Ding X, Wu L, Wu H, Gao X, Borriss R (2018). *Bacillus velezensis* FZB42 in 2018: the gram-positive model strain for plant growth promotion and biocontrol. Front Microbiol.

[5] Nordmann B, Schilling T, Hoppert M, Hertel R (2019). Complete genome sequence of the virus isolate vB_BthM-Goe5 infecting *Bacillus thuringiensis*. Arch Virol.

[6] Ackermann H-W, Prangishvili D (2012). Prokaryote viruses studied by electron microscopy. Arch Virol.

[7] Klumpp J, Lavigne R, Loessner MJ, Ackermann HW (2010). The SPO1-related bacteriophages. Arch Virol.

[8] Bankevich A, Nurk S, Antipov D, Gurevich AA, Dvorkin M, Kulikov AS, Lesin VM, Nikolenko SI, Pham S, Prjibelski AD (2012). SPAdes: a new genome assembly algorithm and its applications to single-cell sequencing. J Comput Biol.

[9] Willms IM, Hoppert M, Hertel R (2017). Characterization of *Bacillus subtilis* Viruses vB_BsuM-Goe2 and vB_BsuM-Goe3. Viruses.

[10] Seemann T (2014). Prokka: rapid prokaryotic genome annotation. Bioinformatics.

[11] Jones P, Binns D, Chang H-Y, Fraser M, Li W, McAnulla C, McWilliam H, Maslen J, Mitchell A, Nuka G (2014). InterProScan 5: genome-scale protein function classification. Bioinformatics.

[12] Ghosh K, Kim K-P (2019). Complete nucleotide sequence analysis of a novel *Bacillus subtilis*-infecting phage, BSP38, possibly belonging to a new genus in the subfamily Spounavirinae. Arch Virol.

[13] Miller SY, Colquhoun JM, Perl AL, Chamakura KR, Kuty Everett GF (2013). Complete genome of *Bacillus subtilis* myophage grass. Genome Announc.

[14] Richter M, Rosselló-Móra R (2009). Shifting the genomic gold standard for the prokaryotic species definition. Proc Natl Acad Sci.

[15] Kurtz S, Phillippy A, Delcher AL, Smoot M, Shumway M, Antonescu C, Salzberg SL (2004). Versatile and open software for comparing large genomes. Genome Biol.

[16] Barylski J, Enault F, Dutilh BE, Schuller MB, Edwards RA, Gillis A, Klumpp J, Knezevic P, Krupovic M, Kuhn JH (2020). Analysis of spounaviruses as a case study for the overdue reclassification of tailed phages. Syst Biol.

[17] Kropinski AM (2009). Measurement of the rate of attachment of bacteriophage to cells. Methods Mol Biol.

[18] Hyman P, Abedon ST (2009). Practical methods for determining phage growth parameters. Methods Mol Biol.

